# Acute Febrile Illness Accompanied by 7th and 12th Cranial Nerve Palsy Due to Lyme Disease Following Travel to Rural Ecuador: A Case Report and Mini-Review

**DOI:** 10.3390/tropicalmed10010021

**Published:** 2025-01-14

**Authors:** Teslin S. Sandstrom, Kumudhavalli Kavanoor Sridhar, Judith Joshi, Ali Aunas, Sheliza Halani, Andrea K. Boggild

**Affiliations:** 1 Division of Medical Microbiology, Department of Laboratory Medicine and Pathobiology, University of Toronto, Toronto, ON M5S 1A8, Canada; 2 Faculty of Arts and Science, University of Toronto, Toronto, ON M5S 3G3, Canada; 3 Division of Infectious Diseases, Department of Medicine, University of Toronto, Toronto, ON M5S 3H2, Canada; 4 Tropical Disease Unit, Toronto General Hospital, Toronto, ON M5G 2C4, Canada; 5 Institute of Medical Science, Temerty Faculty of Medicine, University of Toronto, Toronto, ON M5S 3K3, Canada; 6 Office of Access and Outreach, Temerty Faculty of Medicine, University of Toronto, Toronto, ON M5S 1A8, Canada

**Keywords:** borreliosis, cranial neuropathy, neuroborreliosis, South America, tick-borne disease

## Abstract

The causative agent of Lyme disease, *Borrelia burgdorferi*, is endemic to Canada, the northeastern United States, northern California, and temperate European regions. It is rarely associated with a travel-related exposure. In this report, we describe a resident of southern Ontario, Canada who developed rash, fever, and cranial nerve VII and XII palsies following a 12 day trip to Ecuador and the Galapagos islands approximately four weeks prior to referral to our center. Comprehensive microbiological work-up was notable for reactive *Borrelia burgdorferi* serology by modified two-tier testing (MTTT), confirming a diagnosis of Lyme disease. This case highlights important teaching points, including the classic clinical presentation of acute Lyme disease with compatible exposure pre-travel in a Lyme-endemic region of Ontario, initial manifestations during travel following acquisition of arthropod bites in Ecuador, and more severe manifestations post-travel. Given the travel history to a South American country in which Lyme disease is exceedingly uncommon, consideration of infections acquired in Ecuador necessitated a broad differential diagnosis and more comprehensive microbiological testing than would have been required in the absence of tropical travel. Additionally, cranial nerve XII involvement is an uncommon feature of Lyme neuroborreliosis, and therefore warranted consideration of an alternative, non-infectious etiology such as stroke or a mass lesion, both of which were excluded in this patient through neuroimaging.

## 1. Introduction

The causative agent of Lyme disease, *Borrelia burgdorferi* sensu lato, is a spirochete bacterium transmitted by ticks of the genus *Ixodes*, in particular *I. scapularis* and *I. pacificus* (North America), *I. ricinus* (Western Europe), and *I. persulcatus* (Eastern Europe and Asia). It is the most frequently reported vector-borne disease in Canada, with over 3000 cases in 2021 [1], and is also endemic in the northeastern United States, in northern California, in Europe, and in Asia [2,3,4].

The most common clinical manifestation of Lyme disease is the classic erythema migrans (EM) skin lesion, which occurs within the early localized phase (approximately 5–30 days) following a tick bite. If left untreated, EM is followed by the early disseminated phase of infection and systemic involvement, including central nervous system (CNS) and/or peripheral nervous system (PNS) disease (also known as “Lyme neuroborreliosis”), cardiac involvement, rheumatologic manifestations, and rarely eye involvement with uveitis or keratitis [5]. A subsequent late disseminated phase of infection can follow, characterized by arthritis especially in the knees, and atrophic skin manifestations (Acrodermatitis chronica atrophicans), which is observed mainly in Europe, as it is caused by *Borrelia afzelii*, which is not present in the Americas [6]. Treatment with first-line agents, including doxycycline, amoxicillin, or cefuroxime, is suggested without confirmatory testing in those who present with a reasonable exposure history and compatible dermatologic findings [3]. Testing at our provincial reference laboratory is performed via modified two-tiered testing (MTTT) that employs an IgG/IgM enzyme-linked immunosorbent assay (ELISA) using a whole cell lysate, followed by further tested (tier 2) on a second IgG/IgM ELISA that specifically targets VlsE1 and pepC10 antigens [7,8].

Due to the regional distribution of the *Ixodes* tick, Lyme disease is rarely considered in the context of travel-related illnesses among those visiting the southern hemisphere. In this report, we describe a case of a 65-year-old man with a history of dyslipidemia on statin therapy, who was referred to our clinic for evaluation of rash, fever, and unilateral facial paralysis following a 12 day trip to Ecuador and the Galapagos Islands, taken approximately 4 weeks earlier.

## 2. Case Presentation

A 65-year-old Canadian-born man who resided in an area of southern Ontario endemic for Lyme disease undertook a 12 day trip to both urban and rural Ecuador, including the Galapagos Islands, approximately four weeks prior to his presentation at our center. He reported that he had felt generally unwell upon arrival to Ecuador on Oct 6th, which he attributed to altitude sickness.

One week into his trip on Oct 12th, he developed three isolated, erythematous lesions distributed over the right lateral chin and left lower back/upper buttock. The lesions over the back of the body were described as non-painful; however, the lesion on the chin was noted to be quite tender. It was suspected that these were bites from either a local insect or arachnid due to the size (~1 cm diameter), general appearance, and rapid resolution, but no causative arthropod was found.

After returning home on Oct 18th, he continued to feel generally unwell, with persistent fatigue, lower extremity myalgias, one isolated episode of fever, and the subsequent development of a classic erythema migrans (EM) rash over the left lower back on Oct 26th. This was described as unique in appearance in comparison to the patient’s prior lesions that developed while travelling in that it was non-painful, non-pruritic, uniformly erythematous, and large (spanning an approximate diameter of 6–8 cm), with a “bulls-eye” appearance.

He presented to a local Emergency Department (ED) for assessment and was provided with a two week course of doxycycline 100 mg twice daily, which was initiated on Oct 27th, as well as a short prescription for hydromorphone due to myalgia. On Nov 2nd, he subsequently developed a right-sided facial droop and a non-tender, right lower quadrant abdominal mass. He returned to the ED where an abdominal ultrasound was performed, showing no obvious hernia, focal lesion, or fluid collection. He was instructed to complete his remaining course of doxycycline; however, due to concern that his symptoms were worsening he sought out specialist referral and was seen in our clinic on Nov 7th.

On assessment, the patient was afebrile and well appearing with the exception of a notable right-sided facial palsy. His cranial nerve (CN) exam was significant for a right-sided CN VII deficit with forehead involvement, as well as a mild CN XII palsy with leftward deviation from the midline on extension of the tongue. His neurologic examination was otherwise unremarkable and without symptoms compatible with meningitis or encephalitis. His abdominal exam revealed an approximately 10 cm × 5 cm right lower quadrant mass that was soft, reducible, and non-tender on palpation, with no other hepatosplenomegaly or masses. His cardiovascular, respiratory, lymphatic, and musculoskeletal examinations were otherwise within normal limits. His dermatologic exam was similarly unremarkable, with complete resolution of his previously described left-sided EM lesion.

Investigations available from the patient’s ED visits demonstrated a normal complete blood count and differential, electrolytes, and creatinine, and elevated (but down-trending) liver enzymes, with alanine aminotransferase (ALT) 42 U/L, aspartate aminotransferase (AST) of 40 U/L, and alkaline phosphatase (ALP) 271 U/L. A broad infectious work up, including blood and urine cultures, respiratory virus PCR, malaria thick and thin blood smear and rapid diagnostic test, and serologic testing for Hepatitis A, B, C, and E, Dengue virus, Chikungunya virus, West Nile virus, Yellow Fever, *Rickettsia* (spotted fever and typhi), and *Trypanosome cruzi*, were negative. Notably, his heterophile antibody testing was positive, and it was suspected that a possible Epstein–Barr virus (EBV) reactivation in the context of his acute probable Lyme disease was responsible for his ongoing fatigue and elevated liver enzymes. *Borrelia burgdorferi* serology had been collected previously and was still pending, though the patient’s illness was fully compatible with Lyme disease.

In light of his CN VII and XII deficits, an urgent computed tomography (CT) scan of the brain was arranged (in discussion with the Neuroradiologist on-call at our center) to evaluate for a possible brainstem and/or pontine stroke. Additional infectious workup, including arbovirus, *Bartonella*, and *Leptospira* serologies, *Babesia* detection, repeat malaria screen, and repeat *Rickettsia*, *Trypanosoma*, and Lyme serologies were ordered. Due to the absence of a headache, a fever, or signs/symptoms compatible with bacterial meningitis, and the high pre-test probability that his CN VII deficit was secondary to Lyme neuroborreliosis in the context of his prior EM rash, a lumbar puncture was deferred. This is in keeping with current recommendations by the Infectious Disease Society of America, that recommend diagnosis of Lyme neuroborreliosis by serum antibody testing [3]. Finally, we recommended that he complete his previously prescribed course of doxycycline given the extremely high pre-test probability of his illness representing a primary tick-borne infectious pathology, such as Lyme disease.

Shortly after his clinic visit, the *Borrelia burgdorferi* serologic testing performed during his first visit to the ED was reported as reactive (tier 1 IgG/IgM ELISA: reactive; tier 2 anti-VlsE1+pepC10 IgG/IgM ELISA: reactive), confirming a diagnosis of Lyme disease. His CT brain scan reassuringly demonstrated no evidence of stroke or other intracranial abnormality, and an MRI brain scan was subsequently ordered to further characterize his CN VII and XII deficits. The remainder of his infectious work up remained negative. Upon receipt of confirmatory testing, he was provided an additional two weeks of doxycycline 100 mg twice daily to complete a four week course.

He was seen in follow up two weeks later, with significant improvement in his CN VII and XII deficits and abdominal distension. His repeat Lyme serology had been reported by this point and was again noted to be positive. Unfortunately, due to the nature of this reference-level test, a total antibody titer was not available to compare to his prior serologic testing. During this appointment, he also shared with us that prior to travel he had spent extensive time outdoors walking his dog in a rural area known to have a high tick burden, further confirming our suspicion that this clinical presentation was in keeping with an acute case of Lyme neuroborreliosis. Although it is possible that his abdominal distension represented a pseudohernia secondary to thoracolumbar radiculopathy [9], an equally likely explanation was opioid-induced constipation. Given his significant and rapid improvement with antibiotics alone, follow-up with additional specialties such as Neurology was deferred and his MRI brain scan (which remained pending at the time) was cancelled.

He reported complete symptom resolution six weeks after his first presentation to clinic (approximately 10 weeks post-symptom onset) and the completion of his full course of antibiotics. He remained clinically well at the 6 month follow-up.

## 3. Discussion

Lyme disease is a multisystem inflammatory process that may manifest with CNS signs and symptoms including meningitis, radiculopathies, and cranial nerve involvement. Lyme neuroborreliosis can occur in up to 15% of patients who do not receive treatment for localized Lyme disease [10]. There are a few different proposed mechanisms of how neurologic involvement develops in patients with Lyme disease, including direct infection, autoimmune mechanisms such as molecular mimicry, direct cytotoxicity of *Borrelia* to neural cells, or host cell secretion of neurotoxins or cytokines in response to spirochete infection [11]. Neurologic complications of Lyme disease include cranial neuropathies such as optic neuropathy (and rarely optic neuritis), trigeminal neuropathy, facial nerve palsy, sensorineural hearing loss, and vestibular neuritis, as well as meningitis and radiculopathies [10,12].

Disease manifestations may also differ depending on geographic region, infectious agent, and vector type. For example, late peripheral neuroborreliosis can be observed in Europe in Acrodermatitis chronica atrophicans, and a distinct post-treatment Lyme disease syndrome (PTLDS) may arise due to a migration of *Borrelia afzelii* from the skin to small nerve fibers resulting in small fiber neuropathy [13]. Baggio–Yoshinari Syndrome—an infectious process with a similar clinical presentation as Lyme borreliosis following transmission of *Borrelia burgdorferi* sensu lato complex by hard Ixodid ticks belonging to the *Amblyomma*, *Rhipicephalus* and *Dermacentor* genera—has been described in Brazil [14]. However, whether this exists as a unique clinical entity has been called into question due to a current lack of validated diagnostic tests in this region [15]. 

The most common cranial nerve palsy identified in this context is that of CN VII, the facial nerve, although involvement of other upper cranial nerves has been sporadically reported, particularly that of CN VIII [12]. Involvement of the lower cranial nerves (CN IX-XII) appears to be exceedingly uncommon, with few case reports identified upon a review of the literature. Two of these articles were available only in Spanish, although English translations of abstracts were available and outlined hypoglossal and vagus nerve involvement in the context of a recent Lyme diagnosis [16,17]. Another report described a case of a 65-year-old man who had been previously treated for Lyme meningitis and later presented with a prolonged course of dysarthria due to hypoglossal nerve mononeuropathy thought to be secondary to his prior CNS disease [18]. Recurrent laryngeal nerve involvement [19,20], simultaneous cranial polyneuropathies [21,22,23], and altered maximum cardiac vagal tone during deep breathing [24] have all been described in the context of Lyme neuroborreliosis. Our case adds to the current body of literature supporting the ability of Lyme neuroborreliosis to manifest as lower cranial nerve deficits, given our patient’s 12th CN palsy, and illustrates the importance of having a high suspicion for Lyme disease in patients presenting with “atypical” cranial nerve palsies, particularly those from endemic areas.

This case additionally demonstrates the importance of considering a broad differential for an atypical cranial nerve exam, which should include both infectious and non-infectious etiologies. Our patient’s CN XII palsy, which had gone unnoticed in prior presentations, along with his age and history of dyslipidemia, raised concern for a pontine stroke and thus necessitated urgent imaging to rule out an acute intracranial process.

In Table 1 we have outlined a broad, albeit non-exhaustive, infectious differential diagnosis for patients presenting with cranial nerve deficits, which compiles information from a number of relevant case reports [25,26,27,28,29,30,31,32,33,34,35,36,37,38,39,40,41,42,43,44,45], clinical studies [46,47,48,49,50], and comprehensive review articles [12,51,52,53,54,55,56,57] on the subject. Nonetheless, neurodegenerative or demyelinating diseases, compressive lesions, trauma, ischemia, and inflammatory disorders (e.g., Takayasu vasculitis, sarcoidosis) remain key non-infectious etiologies that should also be considered in the context of isolated or multiple cranial nerve palsies [58]. In the evaluation of these patients, a standardized approach should include a review of epidemiologic risk factors, a thorough physical exam, CT or magnetic resonance imaging (MRI), subsequent infectious work up with input from consulting Infectious Diseases and/or Medical Microbiology colleagues, as well as specialist referral (e.g., Neurology) to facilitate the additional investigation of non-infectious processes, particularly if rapid improvement does not occur with anti-microbial therapy alone.

As a final learning point, this case highlights the importance of considering pre-travel exposures when investigating an infectious cause for illness during or following travel. Although our patient’s symptoms emerged while travelling in rural Ecuador in the context of locally acquired arthropod bites, the likelihood of contracting Lyme disease in that region is exceedingly low given the rarity of *Borrelia burgdorferi* sensu lato in South America [59,60]. In Ecuador specifically, there has only been one reported case of Lyme disease to date, which occurred in a 12-year-old Ecuadorian child [61]. Nonetheless, the dynamic nature of climate change has begun to alter the distribution of vector-borne diseases, and the expanding range of the *Ixodes* ticks should necessitate testing for Lyme disease as well as other tick-borne diseases in individuals traveling from or residing in previously non-endemic areas within the northern hemisphere, who present to care with compatible symptoms [62,63]. By integrating insights from epidemiological research and climate science, healthcare professionals can adopt a proactive approach to disease surveillance and prevention, while addressing emerging health challenges posed by environmental changes and vector-borne diseases.

## 4. Conclusions

In conclusion, this case highlights the need for clinicians to consider a broad differential diagnosis when encountering ill returned travelers with multi-system disease and multiple potential contributory pre-travel and travel-related exposures. Our patient had compelling evidence of pre-travel tick exposure in an area of southern Ontario known to be endemic for Lyme disease. On the other hand, he manifested symptoms approximately one week into his tropical travel in the context of antecedent arthropod bites acquired in Ecuador. Moreover, he presented upon return with the non-specific symptoms of malaise, fever, myalgia, and biochemical hepatitis, all of which warranted exclusion of potentially life-threatening travel-acquired diseases such as malaria, viral hepatitis, and bacteremia. Although he presented with classic clinical features of Lyme disease including a history of EM and new-onset 7th cranial nerve palsy, he additionally presented with atypical features including a 12th cranial nerve palsy. In the context of his age and co-morbidities, this finding alone necessitated consideration of non-infectious intercurrent diagnoses such as stroke. This patient’s entire history and clinical evolution serve as a reminder to clinicians caring for ill returned travelers that while tropical infectious diseases need to be considered and excluded in this population, cosmopolitan infections and illnesses are equally important contributors to the differential diagnosis.

## Figures and Tables

**Table 1:** Differential diagnosis of isolated cranial nerve palsies.

Cranial Nerve	Function	Infectious Differential for Acute Palsy
**CN I** **(Olfactory)**	Sense of Smell	**Viral:** HIV, SARS-CoV-2
**CN II** **(Optic)**	Vision	**Bacterial:** Brucellosis, Lyme disease, *Mycoplasma pneumoniae*, *Mycobacterium tuberculosis*, septic cavernous sinus thrombosis, Syphilis **Viral:** Chikungunya, Coxsackie B, Dengue, EBV, Hepatitis B, HSV, Influenza A, Mumps, Rabies, VZV **Fungal:** Aspergillosis, Blastomycosis, Cryptococcal meningitis, Histoplasmosis, Mucormycosis **Parasitic:** *Baylisascaris procyonis*, *Toxoplasma gondii*, *Toxocara canis/cati*
**CN III** **(Oculomotor)**	Eye movement, Pupil constriction, Eyelid elevation	**Bacterial:** Brucellosis, *Mycoplasma pneumoniae*, *Mycobacterium tuberculosis*, Syphilis, Listeriosis **Viral:** HIV, Rabies, VZV, Zika **Fungal:** Mucormycosis **Parasitic:** *Taenia solium*
**CN IV** **(Trochlear)**	Downward and inward eye movement	**Bacterial:***Mycobacterium tuberculosis*, Syphilis, Listeriosis **Viral:** HIV, Rabies, VZV, Zika **Fungal:** Aspergillosis, Mucormycosis **Parasitic:** *Taenia solium*, *Toxoplasma gondii*
**CN V** **(Trigeminal)**	Sensation to face, chewing muscles	**Bacterial:***Mycobacterium leprae*, Listeriosis, Lyme disease, Syphilis **Viral:** Poliomyelitis, Rabies, VZV **Fungal:** Mucormycosis **Parasitic:** *Toxoplasma gondii*
**CN VI** **(Abducens)**	Lateral eye movement	**Bacterial Infections:** Brucellosis, Listeriosis, Complicated otitis media, Lyme disease, *Mycobacterium tuberculosis*, Syphilis **Viral:** HIV, VZV, Zika **Fungal:** Cryptococcal meningitis **Parasitic:** *Plasmodium falciparum*
**CN VII** **(Facial)**	Facial movement, Taste sensation, Lacrimation, Salivation	**Bacterial:** Brucellosis, Listeriosis, Lyme disease, *Mycobacterium leprae*, *Mycobacterium tuberculosis*, Syphilis **Viral:** EBV, HIV, HSV, Mumps, Poliomyelitis, VZV, Zika **Fungal:** Mucormycosis **Parasitic:** *Toxoplasma gondii*
**CN VIII (Vestibulo-cochlear)**	Hearing, Balance	**Bacterial:** Brucellosis, Lyme disease, *Mycobacterium tuberculosis*, Syphilis **Viral:** Adenovirus, Herpesviridae (including CMV, EBV, HSV, and VZV), Rubella
**CN IX** **(Glosso-pharyngeal)**	Taste sensation, Swallowing, Gag reflex	**Bacterial:***Corynebacterium diphtheriae*, *Clostridium tetani*, Complicated otitis media, *Mycobacterium tuberculosis* **Viral:** HIV, Rabies, VZV, Poliomyelitis **Parasitic/Protozoan:** *Toxoplasma gondii*
**CN X** **(Vagus)**	Swallowing, Autonomic functions, Sensation to throat	**Bacterial:***Corynebacterium diphtheriae*, *Clostridium tetani*, *Clostridium botulinum*, Complicated otitis media, Lyme disease, *Mycobacterium tuberculosis*, Poliomyelitis, Syphilis **Viral:** HIV, Rabies, VZV **Parasitic/Protozoan:** *Toxoplasma gondii*
**CN XI** **(Accessory)**	Shoulder shrug, Head rotation	**Bacterial:***Clostridium tetani*, *Mycobacterium tuberculosis* **Viral:** Dengue, Poliomyelitis
**CN XII** **(Hypoglossal)**	Tongue movement	**Bacterial:***Clostridium tetani*, Lyme disease, *Mycobacterium tuberculosis* **Viral:** Influenza B, HIV, Poliomyelitis

Abbreviations: CMV, cytomegalovirus; EBV, Epstein–Barr virus; HIV, human immunodeficiency virus; HSV, herpes simplex virus; SARS-CoV-2, severe acute respiratory syndrome coronavirus 2; VZV, varicella zoster virus.

## Data Availability

All available data are contained in the report.
